# Evolution of a laboratory mechanomyograph

**DOI:** 10.1007/s10877-024-01175-w

**Published:** 2024-05-17

**Authors:** Zain Wedemeyer, Andrew Bowdle, Srdjan Jelacic, Aidan Lopez, Willis Silliman, Kelly E. Michaelsen

**Affiliations:** Department of Anesthesiology, University of Washington, Box 356540, 1959 NE Pacific Street, Seattle, WA 98195-6540 USA

**Keywords:** Mechanomyography, Quantitative monitoring, Neuromuscular blockade monitoring, Train-of-four monitoring, Medical devices, Twitch monitoring

## Abstract

Mechanomyography is currently the accepted laboratory reference standard for quantitative neuromuscular blockade monitoring. Mechanomyographs are not commercially available. Previously, a mechanomyograph was built by our laboratory and used in several clinical studies. It was subsequently redesigned to improve its usability and functionality and to accommodate a wider range of hand sizes and shapes using an iterative design process. Each version of the redesigned device was initially tested for usability and functionality in the lab with the investigators as subjects without electrical stimulation. The redesigned devices were then assessed on patients undergoing elective surgery under general anesthesia without neuromuscular blocking drugs. Since the patients were not paralyzed, the expected train-of-four ratio was 1.0. The device accuracy and precision were represented by the train-of-four ratio mean and standard deviation. If issues with the device’s useability or functionality were discovered, changes were made, and the redesign processes repeated. The final mechanomyograph design was used to collect 2,362 train-of-four ratios from 21 patients. The mean and standard deviation of the train-of-four ratios were 0.99 ± 0.030. Additionally, the final mechanomyograph design was easier to use and adjust than the original design and fit a wider range of hand sizes. The final design also reduced the frequency of adjustments and the time needed for adjustments, facilitating data collection during a surgical procedure.

**Supplementary Information:**

The online version contains supplementary material available at 10.1007/s10877-024-01175-w.

## Introduction

Due to issues with overshoot with acceleromyography and challenges with electrical noise management with electromyography [1, 2], mechanomyography is currently the accepted laboratory reference standard for quantitative neuromuscular blockade monitoring [3–9]. However, mechanomyographs are not commercially available. Previously a mechanomyograph was built by our laboratory and utilized in several comparison studies of various acceleromyographs, electromyographs, and an archival mechanomyograph [4, 5, 8, 10]. While the usability and functionality of the original design of the mechanomyograph was adequate, it had several limitations. It was difficult to adjust and did not fit all hand shapes and sizes easily, sometimes precluding baseline data collection before administration of neuromuscular blocking drugs. We redesigned our mechanomyograph to improve its usability and functionality.

Mechanomyograph-based quantitative neuromuscular monitoring measures the isometric force exerted by the adductor pollicis muscle [11]. A passive 200-gram force (preload) is conventionally added to the thumb prior to twitch measurement to ensure isometric conditions [11]. The thumb abduction angle needed to maintain the necessary preload varies since the thumb flexibility and size are variable among patients. When using the mechanomyograph on patients undergoing surgery, the arm with the device is frequently repositioned and jostled by the operating room staff, moving the thumb out of position. Additionally, thumb flexibility changes over the course of the surgery as the length of the adductor pollicis brevis muscle increases under constant preload [12, 13]. Therefore, adjustment of the thumb abduction angle may be necessary throughout the surgery to maintain the set preload, which can be distracting for the surgical team. Adjusting the thumb can also be difficult for the researcher as the thumb is often visually and physically obstructed by drapes and surgical equipment.

## Methods

### Redesign process

To address issues found in the original design, an iterative design process was used involving several design versions. We used computer-aided design software Onshape (PTC, Boston, MA, USA) to create the device designs which were then 3D printed using the Original Prusa i3 MK3 Research 3D printer (Prusa Research, Prague, Czech Republic) with polylactic acid plastic (PLA) filament. Hex button head metric M3 screws of varying length (6–35 mm) and M3 nuts were used to hold the device together. Each new version of the design was tested for usability and functionality using first the investigators as subjects in a laboratory setting and then patients as subjects in a clinical setting if the design was deemed satisfactory by investigators in the laboratory setting. If issues with the device’s useability or functionality were found during testing, the redesign process was repeated. Unlike the testing in the clinical setting, the testing in the laboratory setting using investigators did not involve the application of electrical stimuli or data collection.

### Human studies testing

The usability and functionality of the redesigned mechanomyograph were compared to the original design by measuring the range of motion, height, and weight of each device. Range of motion was defined as the maximum minus the minimum angle achievable by the preload adjustment mechanism. Height was measured perpendicular from the base to the highest point on the thumb immobilizer. The base is the part of the thumb immobilizer that was secured to the wrist brace parallel to the forearm. Weight of the assembled 3D printed parts not including the force transducer or the wrist brace was measured.

Mechanomyograph accuracy and precision were determined by measuring train-of-four ratio using the redesigned version of the mechanomyograph in patients undergoing elective surgery under general anesthesia. Patients were enrolled if they were greater than 18 years of age, received general anesthesia without neuromuscular blocking drugs, and had at least one arm accessible during surgery. Patients with known neuromuscular abnormalities were excluded. All patients gave written informed consent. This study was approved by the University of Washington institutional review board and registered on ClinicalTrials.gov as NCT05006807 on 8.16.21.

Twenty-five patients were enrolled (an interim version was used in 4 patients and the final version was used in 21 patients). An electromyograph and the redesigned mechanomyograph were placed on the same arm of each patient. The electromyograph was used to stimulate the patient’s ulnar nerve while the mechanomyograph recorded data from the force transducer as previously described [10]. Preload was determined in the laboratory setting by measuring the voltage at the limits of the recommended preload range of 200 and 300 g using corresponding calibration weights as previously described [10]. Investigators verified that preload was maintained during data collection in the clinical setting by verifying that the transducer voltage output was in the expected range which was displayed in real-time on the computer monitor that interfaced with the transducer.

Train-of-four ratios were measured every 15 s for the duration of the anesthetic for each patient. Because patients did not receive neuromuscular blocking agents, the expected train-of-four ratio was 1.0 [14]. Accuracy is defined as how close the mean train-of-four ratio is to 1.0. Precision is defined as repeatability, represented by the standard deviation of the train-of-four ratio. A limited data set of 6 patients who received neuromuscular blocking drugs was also recorded the same way as described above for the patient study. However, measurements occurred less frequently than every 15 s due to lengthy periods of deep neuromuscular blockade.

## Results

### Original design

**Fig. 1:**
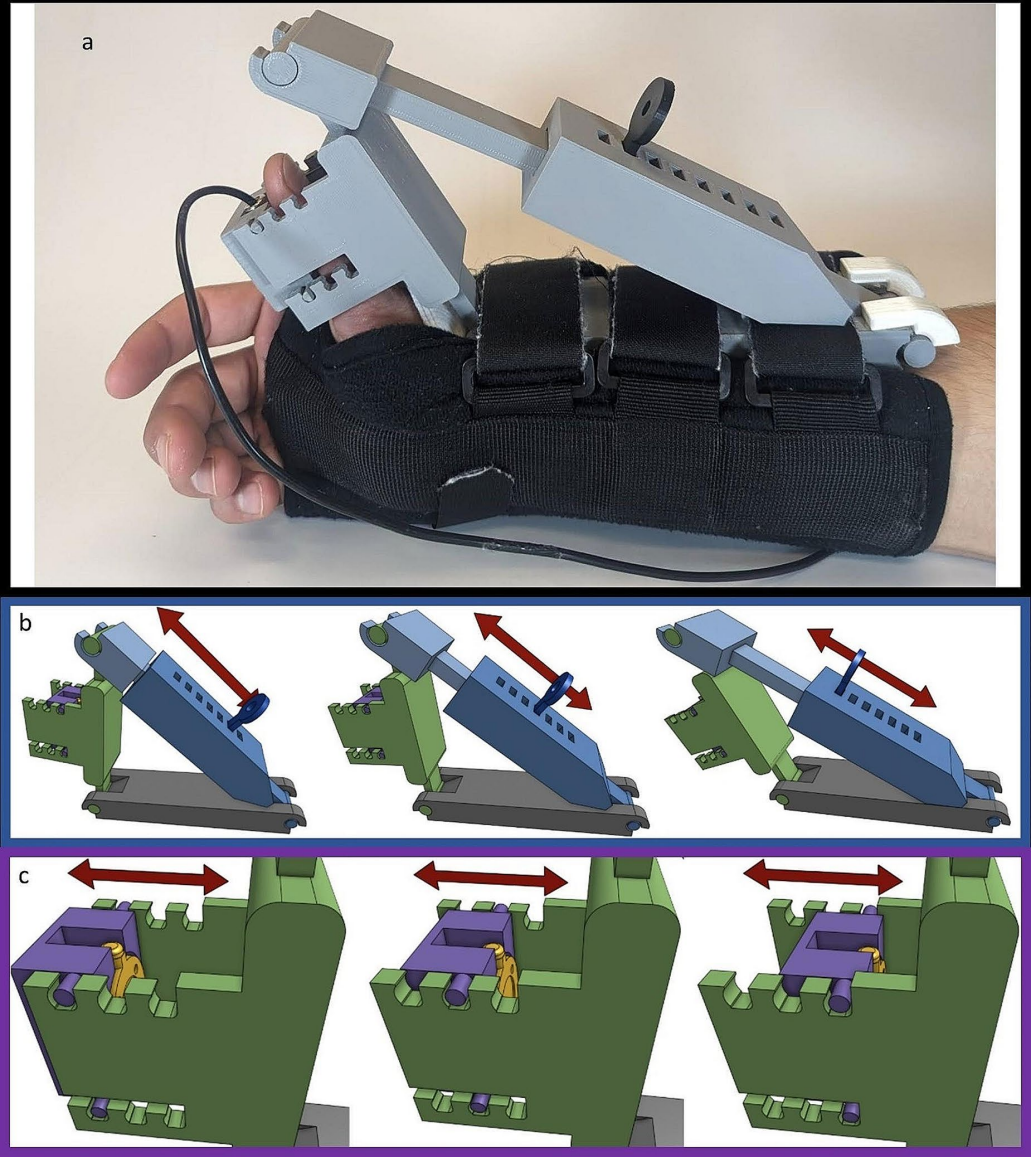
Original design mechanomyograph. **a** Picture of the original design mechanomyograph as it would be used on a patient. **b-c** Screenshots of the 3D model. Individual parts in panels **b** and **c** represented by different colors are printed separately and then assembled. They are colored in the illustration for demonstration purposes and do not reflect the actual device color shown in **a**. **b** Three different positions attainable using the main preload adjustment mechanism shown in blue. **c** Three different positions attainable using the secondary preload adjustment mechanism shown in purple and green. Red arrows show direction of movement of the adjacent mechanisms

The original design of our mechanomyograph used a custom 3D printed adjustable thumb immobilizer fastened to a commercial wrist brace (Universal Wrist/Forearm Splint, DonJoy, Lewisville, TX, USA) (Fig. 1) as previously described [10]. The plastic thumb immobilizer affixed a button load cell force transducer (CB9 100 N, HBM, Darmstadt, Germany) directly underneath the distal phalanx of the thumb while the wrist brace secured the immobilizer to the patient and prevented wrist movement.

The original design had several issues, including less than optimal adjustability, range of motion and lack of thumb length adjustment. Two slot and key style mechanisms were used to adjust the thumb abduction angle which required two hands to operate and contained small loose parts that were easily dropped. There was no adjustment system to accommodate different thumb lengths. Additionally, the range of motion of the thumb abduction angle adjustment system was too limited to maintain enough preload in patients with highly flexible thumbs. When the original design did not easily fit a patient’s hand, various amounts of gauze padding were placed between the plastic thumb immobilizer and the wrist to adjust the force transducer position until it was underneath the thumb. Although this solution allowed the device to fit on different hand sizes and shapes, it required a trial-and-error process to place gauze correctly, was prone to displacement, and was time-consuming.

To accommodate for these issues, the user needed frequent, unobstructed access to the device and both hands to easily set up and maintain the appropriate force transducer position and preload. This was difficult in a busy operating room with surgical equipment and care team members surrounding the patient. Researchers often crawled underneath equipment and crouched near the device for several minutes to make the necessary adjustments without disturbing the surgical team.

### Redesign version 1

**Fig. 2:**
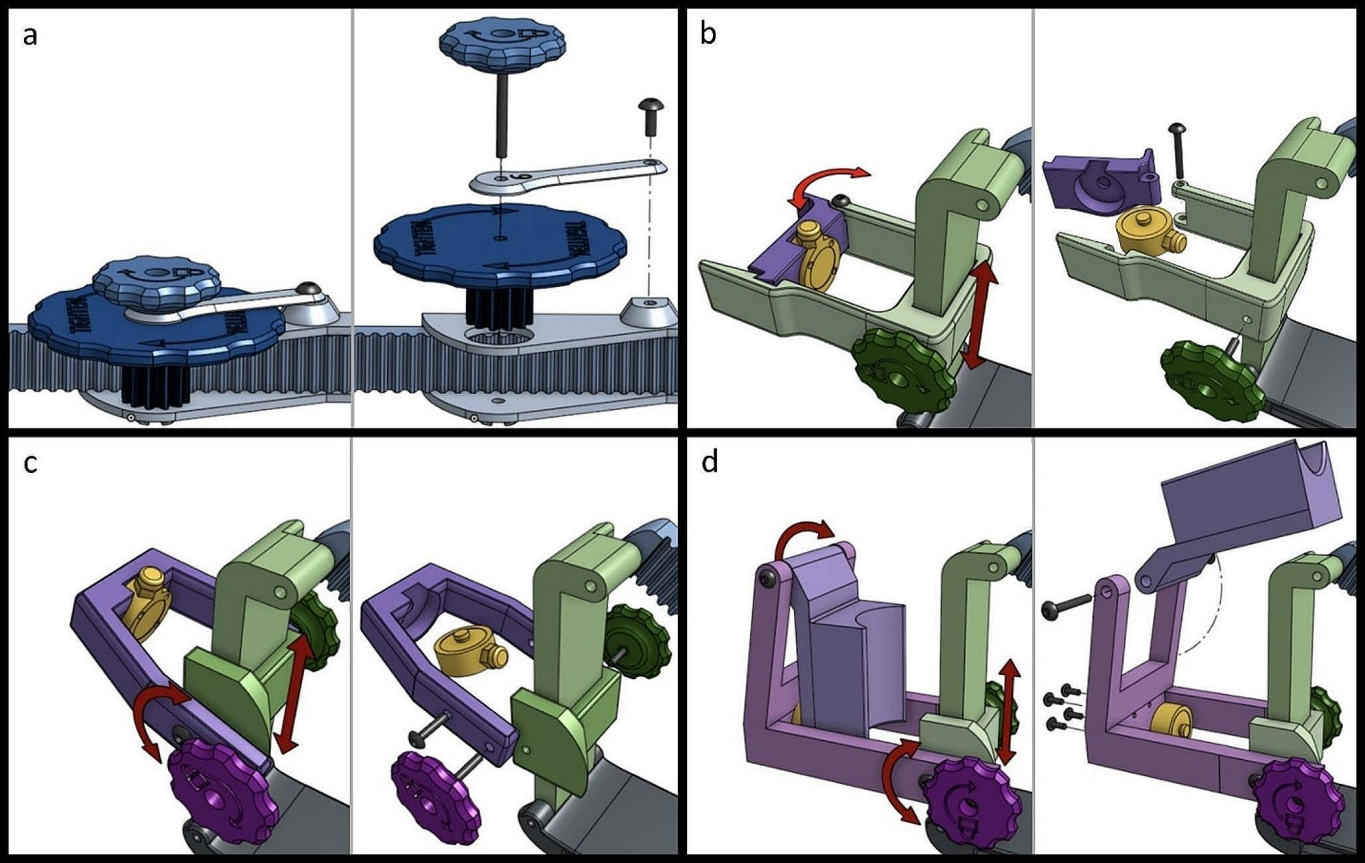
Screenshots from the 3D models of sub-assemblies at various stages in the iteration process. Each panel shows an assembled view (left) with red arrows indicating movement, and an exploded view (right). **a** Preload adjustment mechanism. **b** Transducer mount used in the redesigned version (1) **c** Transducer mount used in the redesigned version (2) **d** Transducer mount used in the final version. Individual parts represented by different colors are printed separately and then assembled. They are colored in the illustration for demonstration purposes and do not reflect the actual device color

The first system to be redesigned was the preload adjustment mechanism. The original design used a key and slot mechanism shown in Fig. 1b. It was replaced with a locking rack and pinion gear system (Fig. 2a).

The new design allowed for continuous adjustment of the preload by turning a large knob. Tightening a secondary smaller knob locked the position of the pinion gear in place. Unlike the original design, the redesigned system was not limited to a set of discrete positions, allowing for finer adjustments to be made. The redesigned system eliminated loose parts that could be dropped and was quickly adjustable with one hand without needing to visualize the device. Maintaining preload with the redesigned mechanomyograph was easier to accomplish without disturbing the surgical team in busy operating rooms.

The transducer mount was also redesigned. Early in the redesign phase, the original force transducer stopped functioning. Rather than replacing it with the exact same model, a smaller version was selected to decrease the overall device size and weight (MCL-01 Miniature Compression Button Load Cell, Load Cell Systems, Towanda, PA, USA). The new force transducer was rated for 0–50 Newtons of force. The original transducer mount consisted of a key and slot mechanism to move the transducer in and out (Fig. 1c). This mechanism was effectively another way to adjust the preload of the device, making it somewhat redundant to the previously described preload adjustment system. It also included a detachable piece, which was useful in allowing the thumb to be quickly inserted into the device. However, the detachable piece could accidentally fall out requiring the research team to search for this piece during clinical care. Also, the device could not be adjusted to accommodate different thumb lengths. The replacement design used a linear sliding mechanism to accommodate for different thumb lengths with a set screw to lock it into position (Fig. 2b). The set screw was integrated with a knob so it could be turned easily. The transducer mount (shown in purple in Fig. 1), was changed to a swinging door mechanism to prevent it from falling out. The door mechanism housed the transducer in a cylindrical cavity similar to the original design (Fig. 2b).

**Fig. 3:**
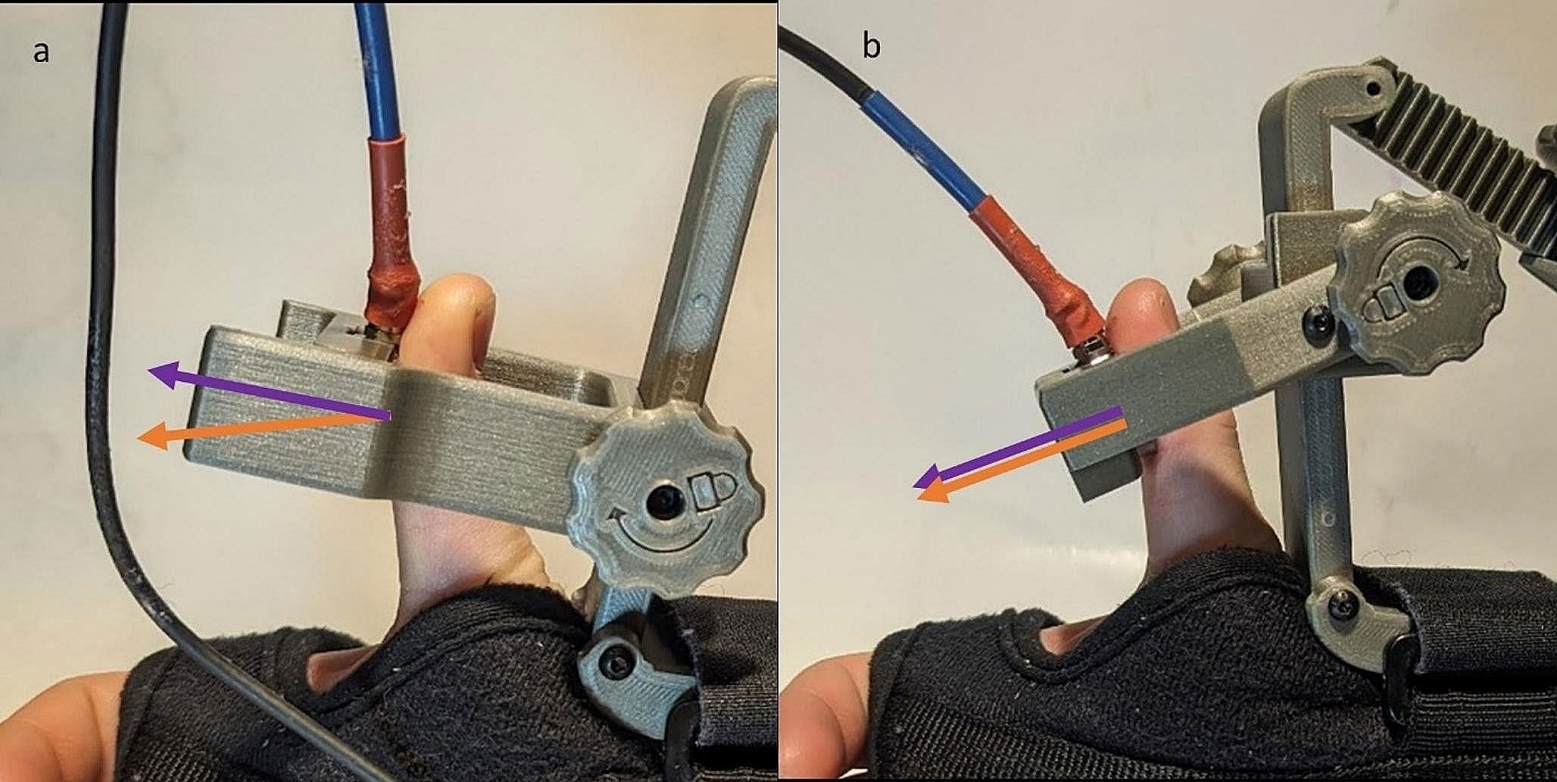
**a** Images of the transducer mount from redesign version 1. **b **Transducer mount from redesign version 2 of the mechanomyograph. Purple arrows show the direction of alignment of the force transducer. Orange arrows show the direction of force exerted by the thumb. Both panels show the thumb at the same angle of abduction

Redesign version 1 was 3D printed, assembled, and tested for usability and functionality in the lab. Several observations were made. First, the swinging door transducer mount was unnecessary as the device could be positioned correctly without opening the door. This was accomplished by sliding the transducer mount to its highest position, positioning the thumb beneath it, then sliding it back down over top of the thumb until the distal phalanx was resting on the force transducer. Second, the force transducer was difficult to position at an angle that maximizes the amount of force transferred from the thumb to the transducer. The transducer only measures force acting on it in a direction perpendicular to its face. Often the thumb could not be aligned perfectly resulting in a component of the thumb force being lost (Fig. 3). Therefore, instead of testing the redesign version 1 in patients, we moved on to redesign version 2.

### Redesign version 2

The design of the transducer mount was modified based on lessons learned from redesign version 1 (Fig. 2c). The swinging door mechanism was eliminated, simplifying the design. The linear sliding mechanism was modified to allow the transducer mount to pivot. A knob was added which would lock the pivoting mechanism in place with a set screw when tightened. This allowed the user to position the transducer in line with the force of the thumb.

**Fig. 4:**
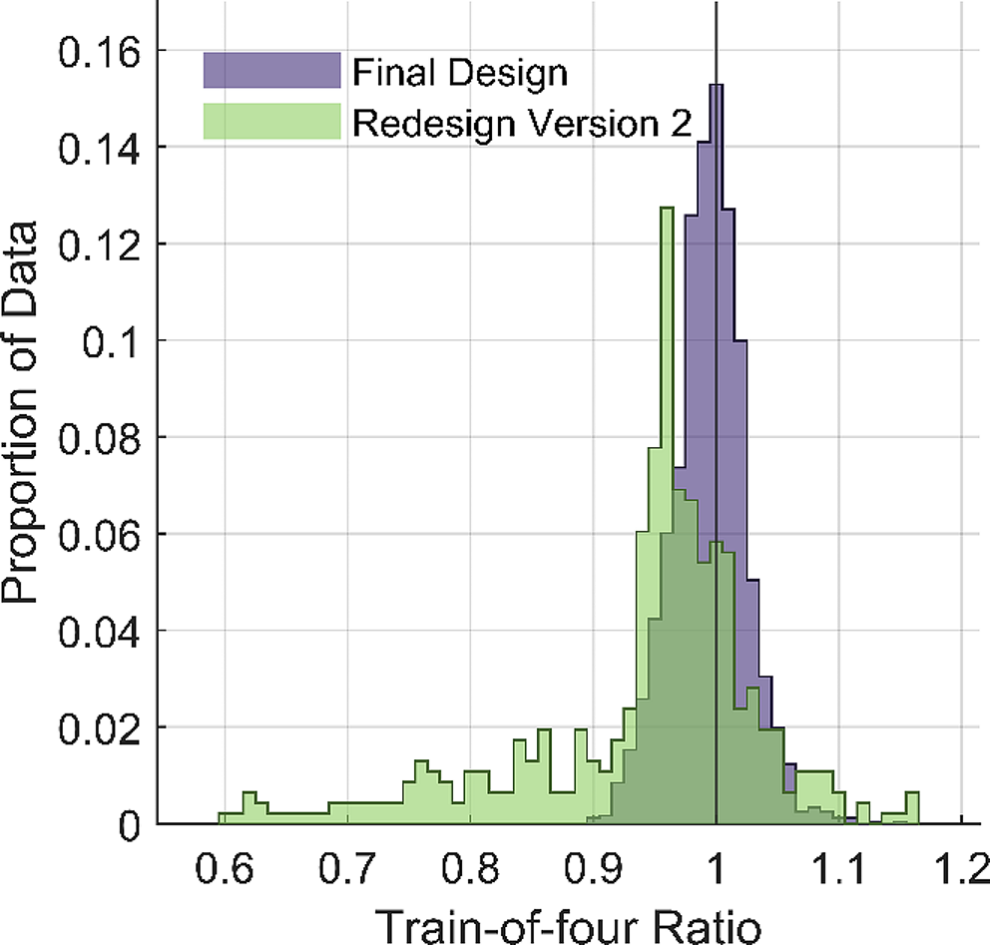
Histogram showing performance data from redesign version 2 and the final design. Bins are a distance of 0.01 apart

After the new design was successfully tested for usability and functionality in the lab, the mechanomyograph’s performance was tested as described in the methods. Four-hundred-sixty-three train-of-four ratios were collected from four patients under general anesthesia without neuromuscular blocking drugs (Table 1; Fig. 4).

**Table 1:** Patient characteristics

Characteristic	Redesign Version 2	Final Design
Number of patients	4	21
Age (years)	63 ± 7 (54–68)	54 ± 18 (24–82)
Sex (F)	3 (75%)	9 (43%)
BMI (kg/m^2^)	30.3 ± 4.1 (25.0–34.9)	28.9 ± 5.2 (19.1–41.6)
Duration of Surgery (minutes)	93 ± 35 (64–143)	90 ± 39 (46–198)
Number of Train-of-four Ratio Measurements per Patient	116 ± 26 (90–151)	112 ± 92 (23–352)

Continuous variables are presented as mean ± standard deviation (range)

The mean train-of-four ratio was 0.94 ± 0.097. After close inspection of the waveforms, the investigators noticed that abnormally low train-of-four ratios (< 0.9) were often associated with irregularly shaped waveforms in select patients without the obvious sources of irregularities such as electrocautery or inadvertent thumb movement. After investigating the transducer and housing cavity, it was discovered that the force transducer could become wedged at a slight angle within the housing cavity after the initial twitch response since the transducer was not fixed in place (Fig. 2b-c). This caused attenuation of the subsequent twitch amplitudes as part of the response to train-of-four stimuli. As a result, the amplitude of T4 was less than T1 causing an artificial decrease in the train-of-four ratio (T4/T1). Once this problem was discovered, patient trials were stopped, and another design iteration began.

### Final design

**Fig. 5:**
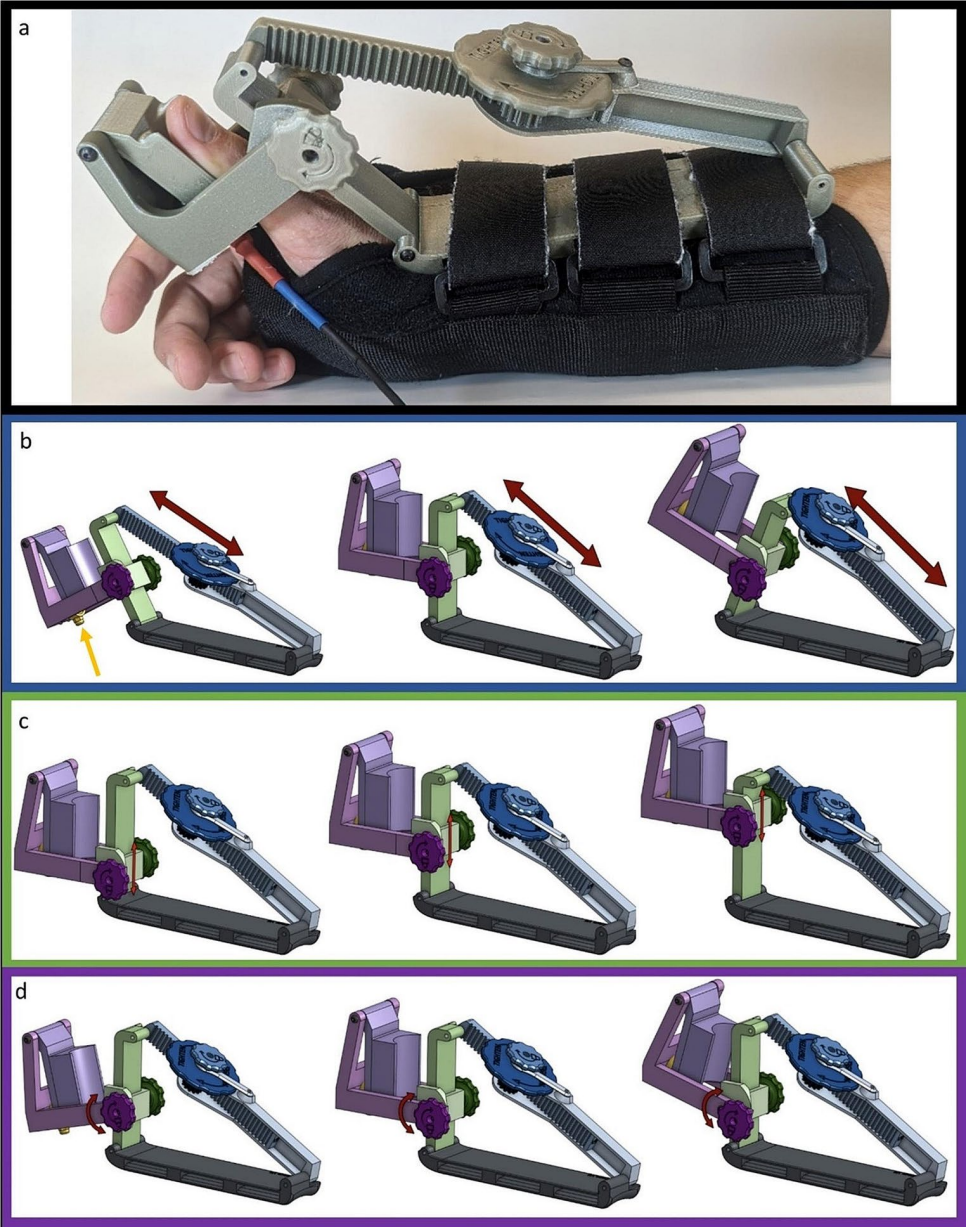
Final design of the mechanomyograph. **a** Image of the device placed as it would be on a patient. **b-d** Three different potential positions of an adjustment mechanism. Direction of movement is indicated with red arrows. Individual parts in **b-d** are printed separately and then assembled. They are colored in the illustration for demonstration purposes and do not reflect the actual device colors shown in **a**. **b** Preload adjustment mechanism in blue. **c** Thumb length adjustment mechanism colored green. **d** Force transducer mount pivoting mechanism in purple. The force transducer is colored yellow. A yellow arrow in **b** points to the force transducer. For a video showing all three adjustment mechanisms in motion, see supplementary information

The transducer mount was redesigned to prevent force transducer movement inside the housing cavity. In the final version, screws were used to fix the transducer firmly in place, preventing any movement. The transducer mounting holes were located on the face of the transducer where contact was made with the thumb in previous versions. In order to use the mounting holes to secure the transducer in place, the transducer was flipped so the button side faced towards the thumb. An additional hinged component was added to transfer the force of the thumb to the button of the transducer as the surface area of the button was too small to measure force from the full area of the thumb (Fig. 2d). The linear sliding mechanism and pivot mechanism remained unchanged. Additionally, slots were added in the base of the thumb immobilizer (shown in grey in Fig. 5) to allow the hook and loop straps of the wrist brace to pass through the base, holding it more securely to the forearm.

The final version’s performance was tested in 21 patients under general anesthesia without neuromuscular blocking drugs (Table 1). Two-thousand-sixty-two train-of-four ratios were collected. The train-of-four ratio mean and standard deviation were 0.99 ± 0.030 (Fig. 4).

**Fig. 6:**
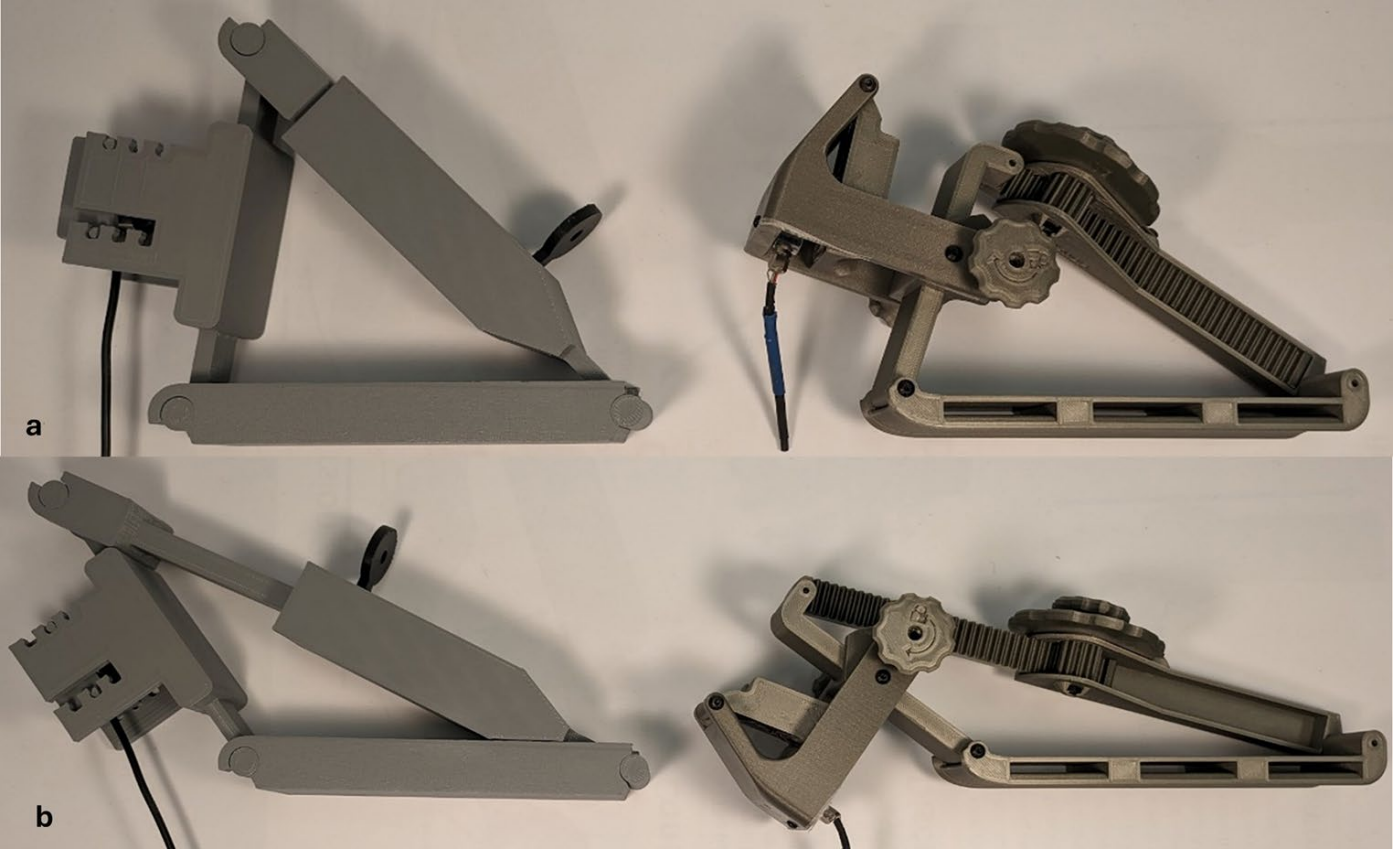
Each panel shows side by side comparison of the original mechanomyograph (left) and the redesigned mechanomyograph (right). **a** shows both mechanomyographs with the preload adjustment mechanism in their fully retracted state. **b** shows both mechanomyographs with the preload adjustment mechanisms in their fully extended state

The final design and the movement of the three adjustment mechanisms are shown in Fig. 5. Throughout the iteration process, an effort was made to make the device smaller, lighter, and more adaptable to different thumbs. When measured, these three metrics improved (Table 2). A side-by-side comparison of the original and final design of the mechanomyograph is shown in Fig. 6.

**Table 2:** Original design versus final design mechanomyograph specifications

Specification	Original design	Final version
Range of Motion	51 degrees	82 degrees
Height	5.6 inches	3.9 inches
Weight	193 g	120 g

## Discussion

The iterative prototyping process described above led to improvements in the mechanomyograph’s precision and accuracy. The final version had a mean train-of-four ratio (0.99) which was nearly the expected value (1.0), and the standard deviation was small (0.030).

Mechanomyography was first used for neuromuscular blockade monitoring in the 1970s [11, 15]. Several mechanomyography-based quantitative neuromuscular monitors were manufactured including the Relaxometer and Myograph 2000 [16, 17]. These devices were used mainly for clinical research and went out of production more than 20 years ago, but until recently were the only devices available. We are only aware of three mechanomyography-based quantitative neuromuscular monitors built during the last 20 years; the one discussed in this paper, another hand built mechanomyograph^1^, and the Isometric Thumb Force© transducer [18–20]. Our original mechanomyograph has been compared to an archival mechanomyograph [10].

Of the 21 patients included in this study with the final version, none were excluded due to poor device functionality or technical difficulties. The final version was adjustable with one hand, allowed for greater range of motion, and was smaller and lighter than the original design, thus, improving functionality and usability. The final version allowed for data collection to be conducted in busy operating rooms without obstructing patient care and leading to more patients being enrolled in a shorter time span than with the original design.

A limitation of this study is that the data used for analysis of accuracy and precision were collected on patients not receiving neuromuscular blocking drugs. The performance of the device in the presence of neuromuscular blocking drugs may differ. An advantage of collecting train-of-four ratio data in the absence of neuromuscular blocking drugs is that the expected train-of-four ratio is always 1.0. Thus, the accuracy and precision of the mechanomyograph train-of-four ratios can be determined through comparison to the expected value. We previously compared train-of-four ratios in patients receiving neuromuscular blocking drugs using the original design of our mechanomyograph to an archival mechanomyograph and showed similar results between the two devices [10]. The original design of our mechanomyograph also performed well when counting twitches in comparison to palpation [5]. A limited data set of train-of-four ratios collected from 6 patients who received neuromuscular blocking drugs is included in the supplementary information, for the purposes of illustrating the function of the mechanomyograph with a wide range of depth of neuromuscular blockade.

The design described here is open source and can be reproduced quicky and cheaply due to its 3D printed design. The material cost of the 3D printed components alone was about $5. Although the specific 3D printer that we used to print these components costs $900, a lower-end 3D printer costing closer to $200 can be used since the components are small and have a simple design. With the additional cost of the wrist brace and screws used for assembly, the total price is about $285. It was printed in less than a day on a consumer grade 3D printer. The 3D model files are available upon request.

## Conclusion

The final design was adjustable with one hand, allowed for a greater range of motion to accommodate a larger range of hand sizes and shapes, and was smaller and lighter than the original design. The improvements to the mechanomyograph combined with open-source access to the design, may encourage more widespread use of mechanomyograph monitoring during studies of neuromuscular blocking drugs and neuromuscular blockade monitors.

## Electronic supplementary material

Below is the link to the electronic supplementary material.

Supplementary Material 1

## Data Availability

No datasets were generated or analysed during the current study.

## Footnotes

1 Roopvir Kaur MD, Julia Vogt BS, Ellie Shibly BS, Zafar Iqbal MD, Sweeta Gandhi MD, Thomas J. Ebert, MD, PhD. Validating Clinically Important Neuromuscular Monitoring Endpoints: Mechanomyography (MMG) vs. Electromyography (EMG). In: Abstracts of International Anesthesia Research Society & Society of Critical Care Anesthesiologists 2023 Annual Meetings. Anesthesia & Analgesia. 2023; 136 (5 S): 1031–1032. 10.1213/01.ane.0000977652.27377.97.
