# Supplementary material for: Evolution of a laboratory mechanomyograph
Source: J Clin Monit Comput. 2024 May 17;38(6):1415–23. doi: 10.1007/s10877-024-01175-w (PMC11604740; doi:10.1007/s10877-024-01175-w)
Supplement: Supplementary file 1 — Supplementary Material 1 [file 10877_2024_1175_MOESM1_ESM.docx]

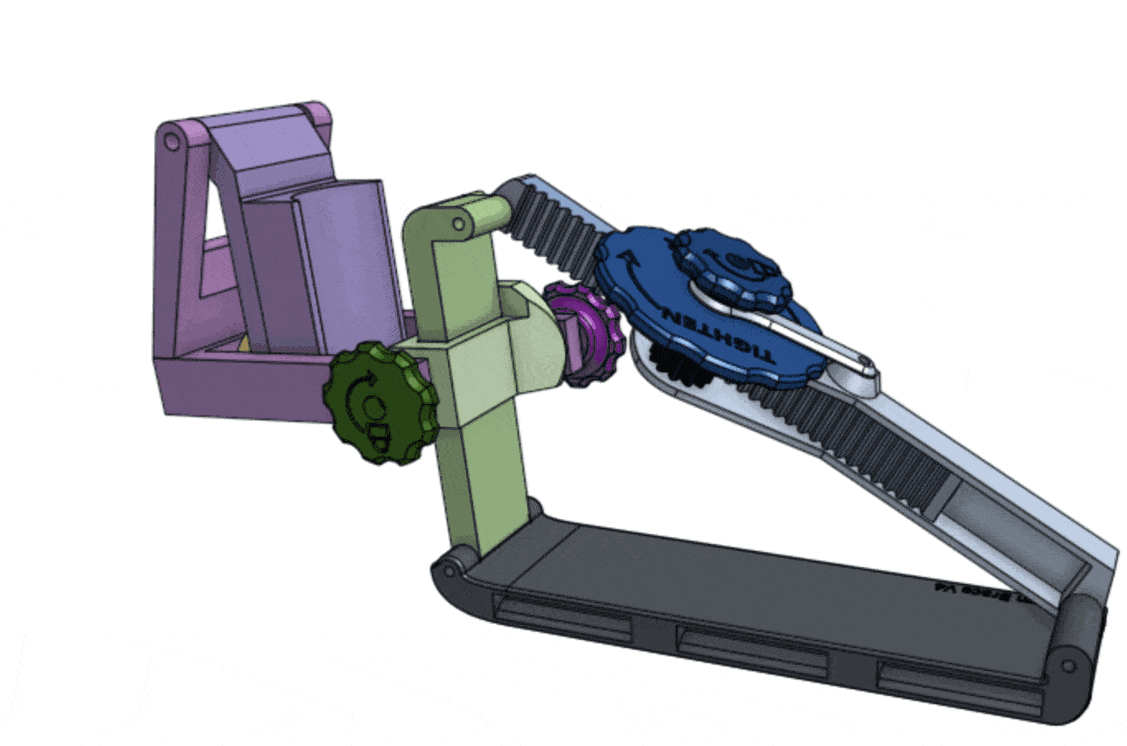


Animation showing the movement of the three adjustment mechanisms in the final version of the mechanomyograph


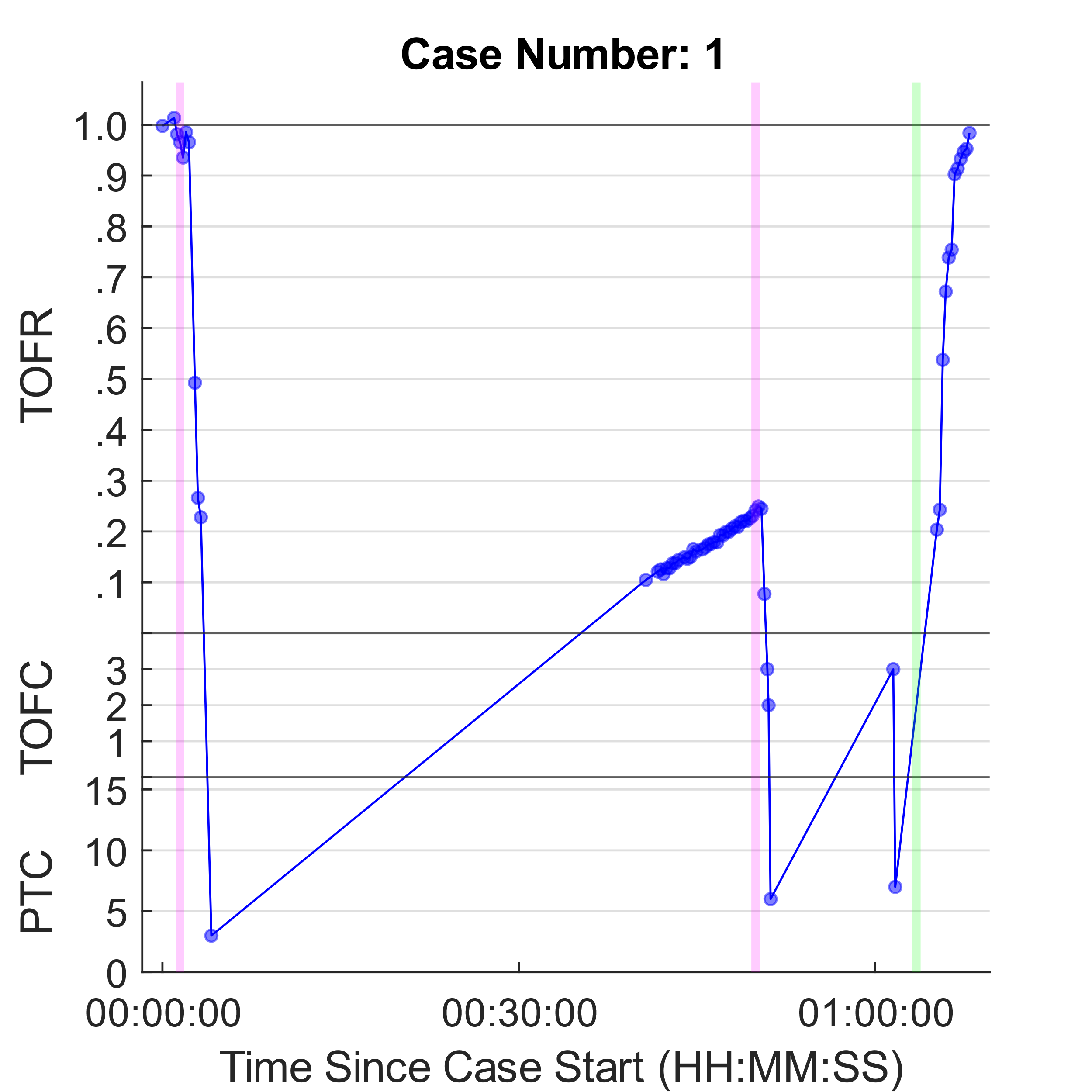

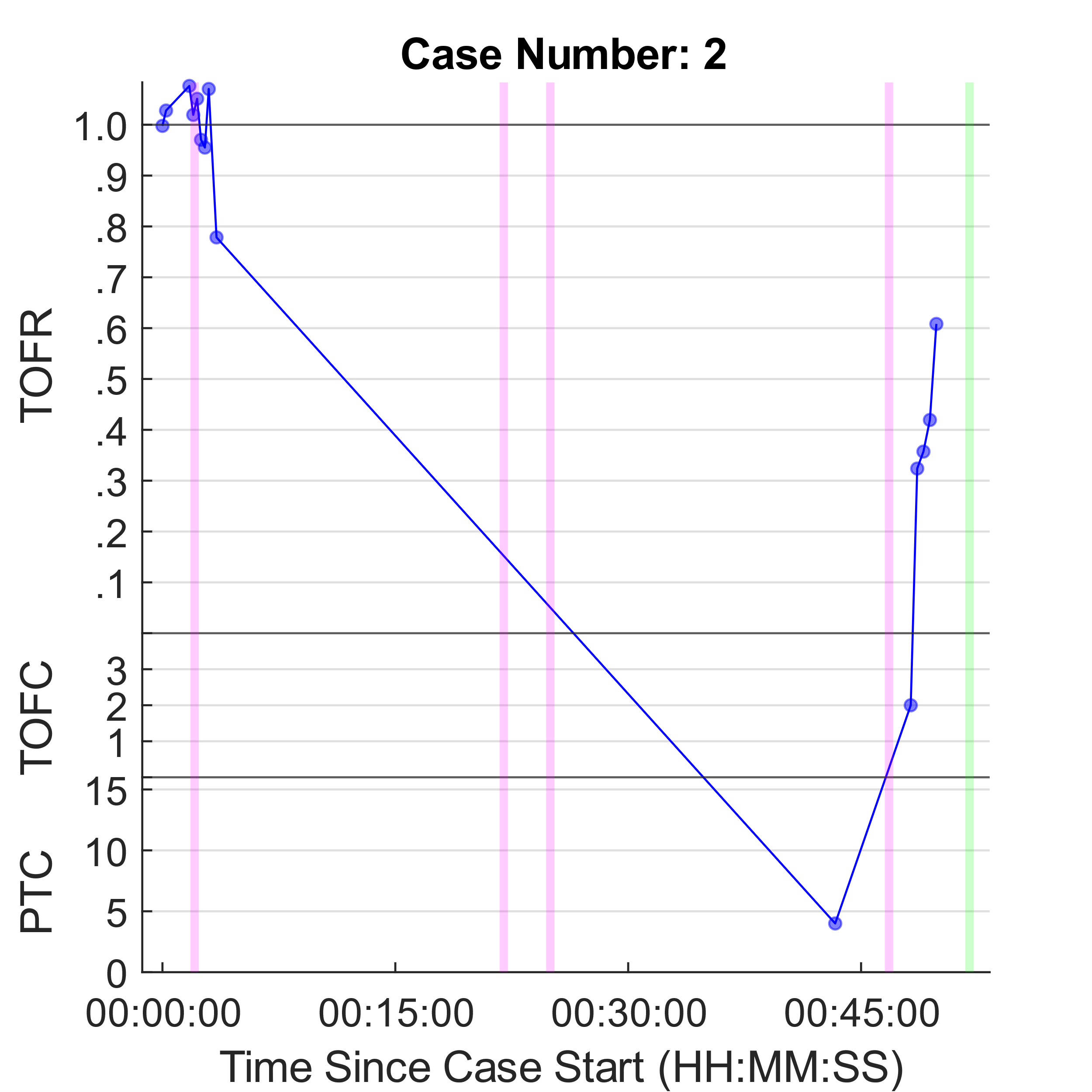

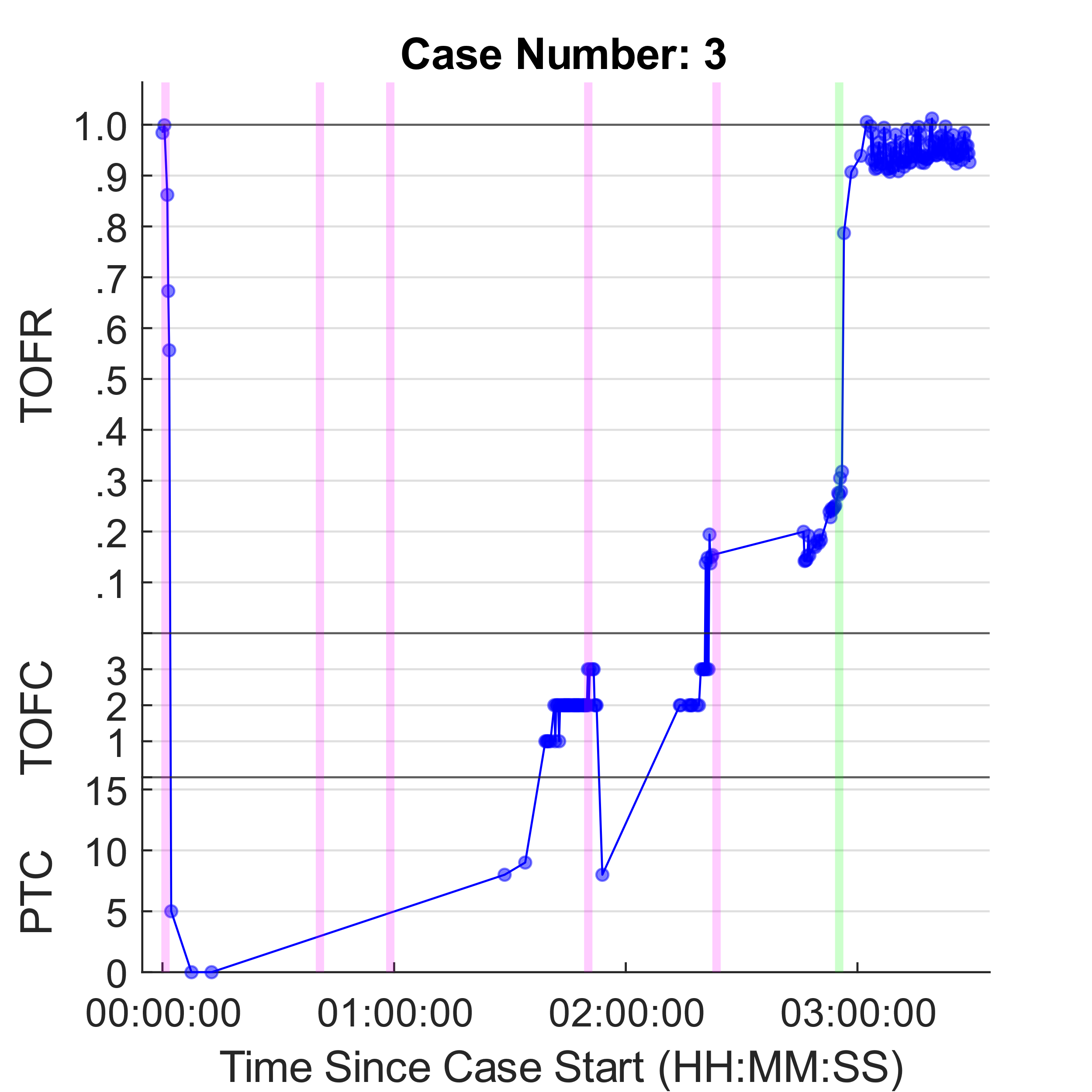

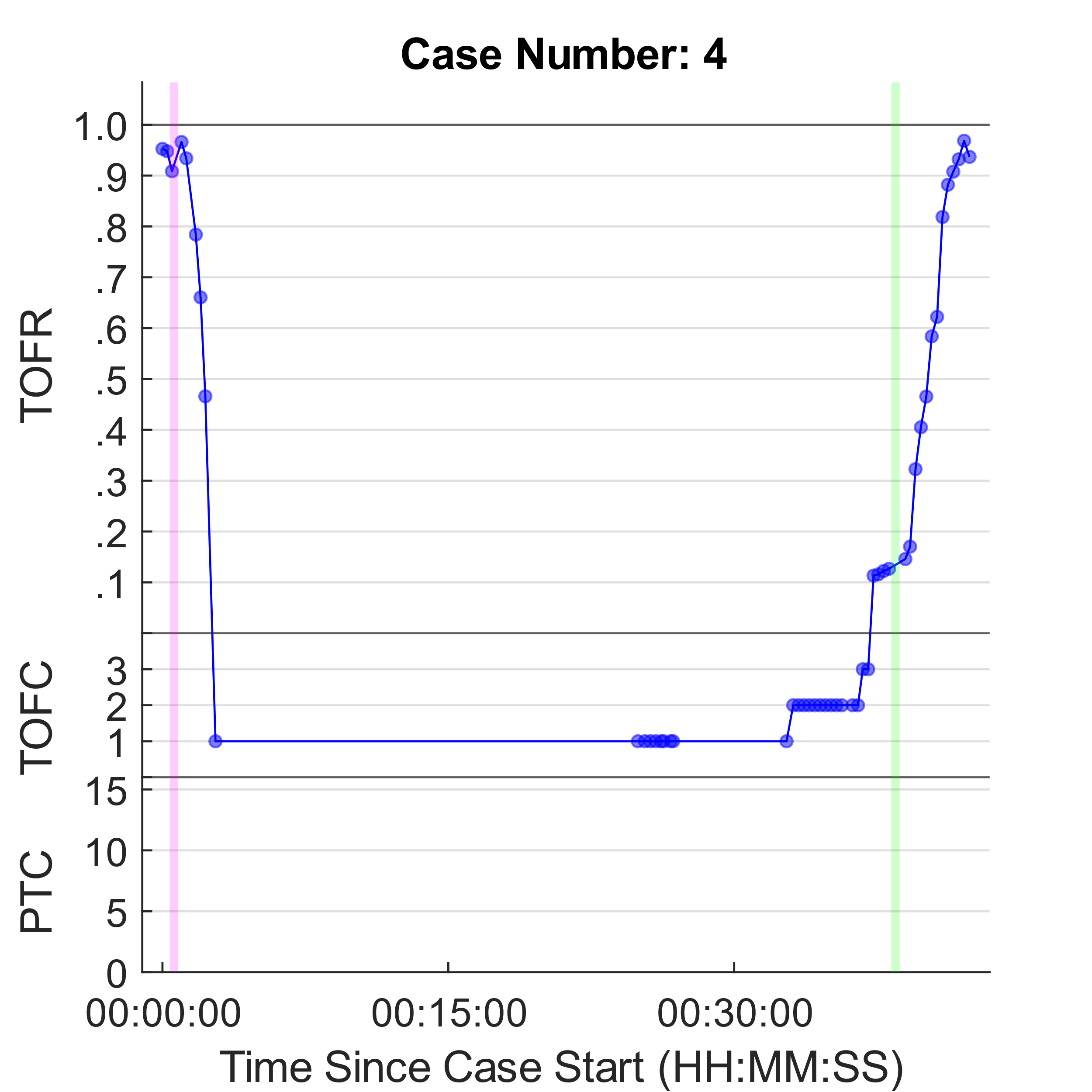

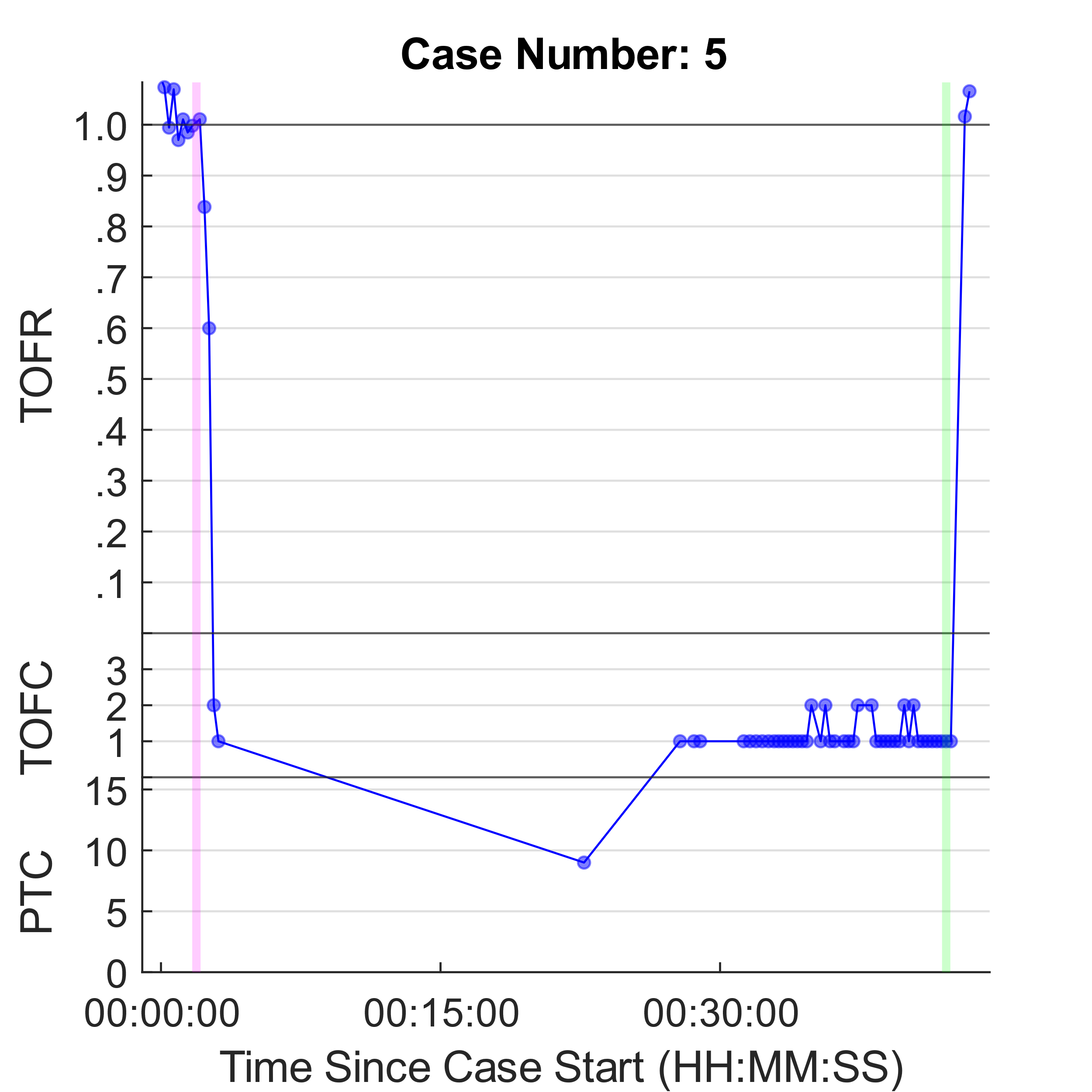

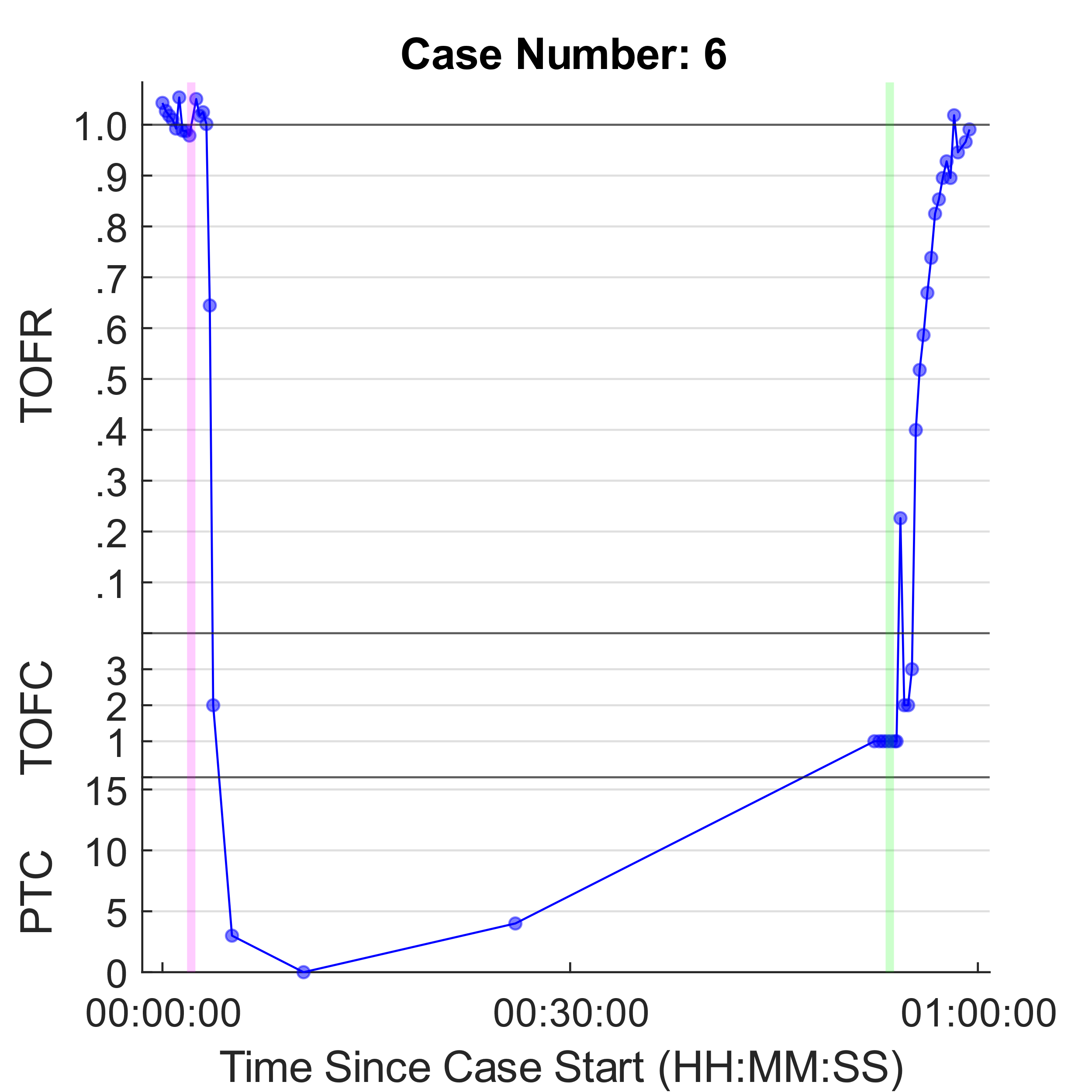


Graphs show the level of neuromuscular block over time measured using the redesigned mechomyograph for six patients receiving neuromuscular blocking drugs. Post tetanic counts (PTC), train-of-four counts (TOFC), and train-of-four ratios (TOFR) are all shown on the y-axis. Magenta vertical lines indicate when rocuronium was administered. Green vertical lines indicate when suggammadex was administered.
